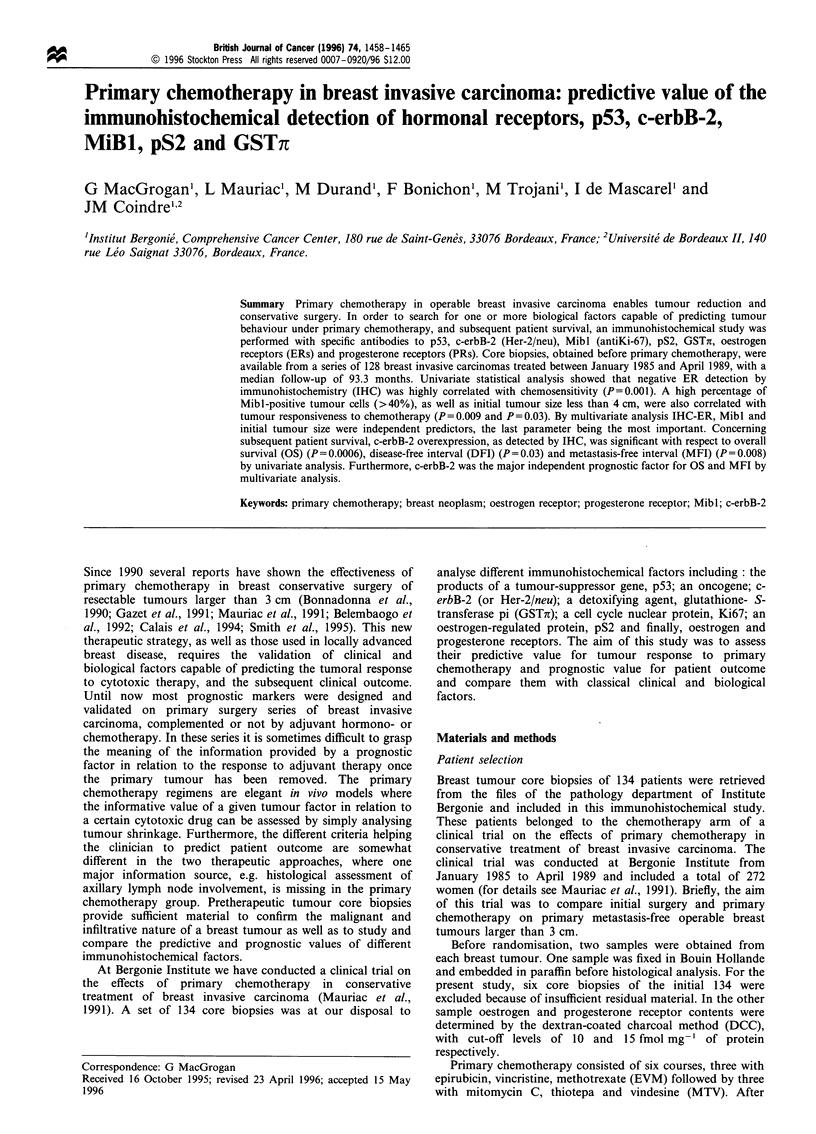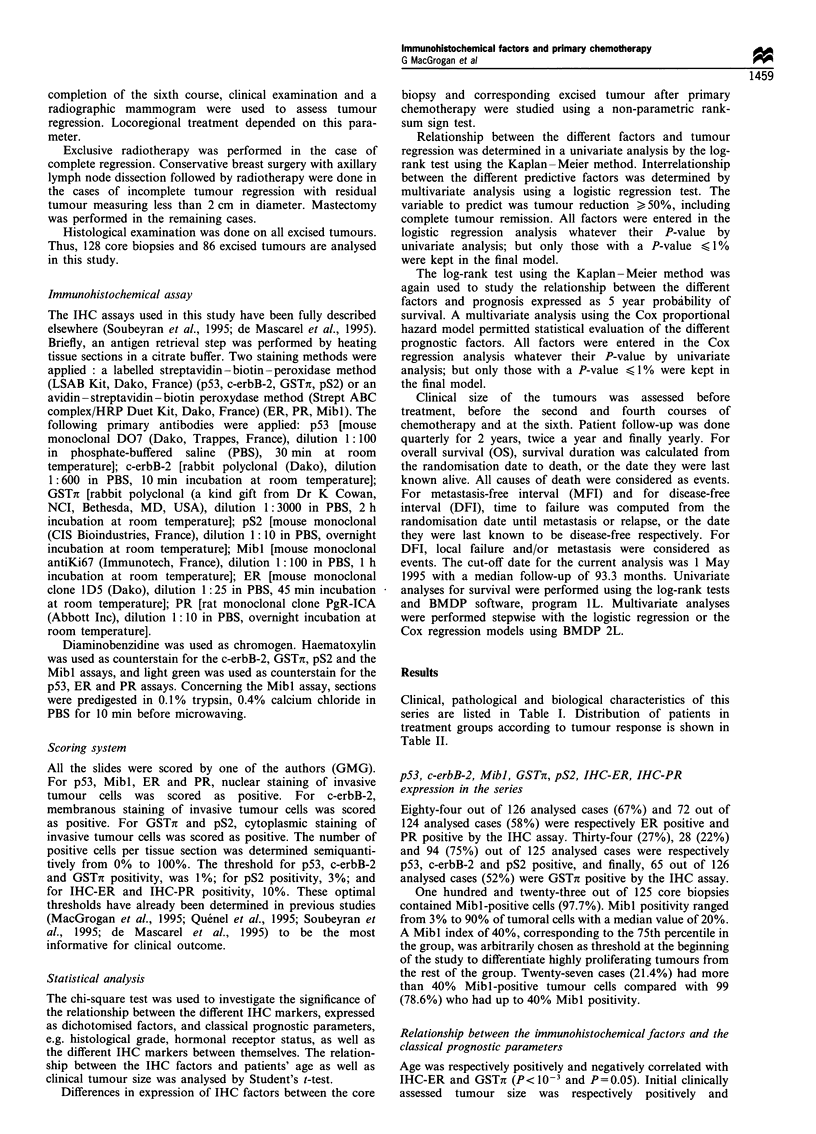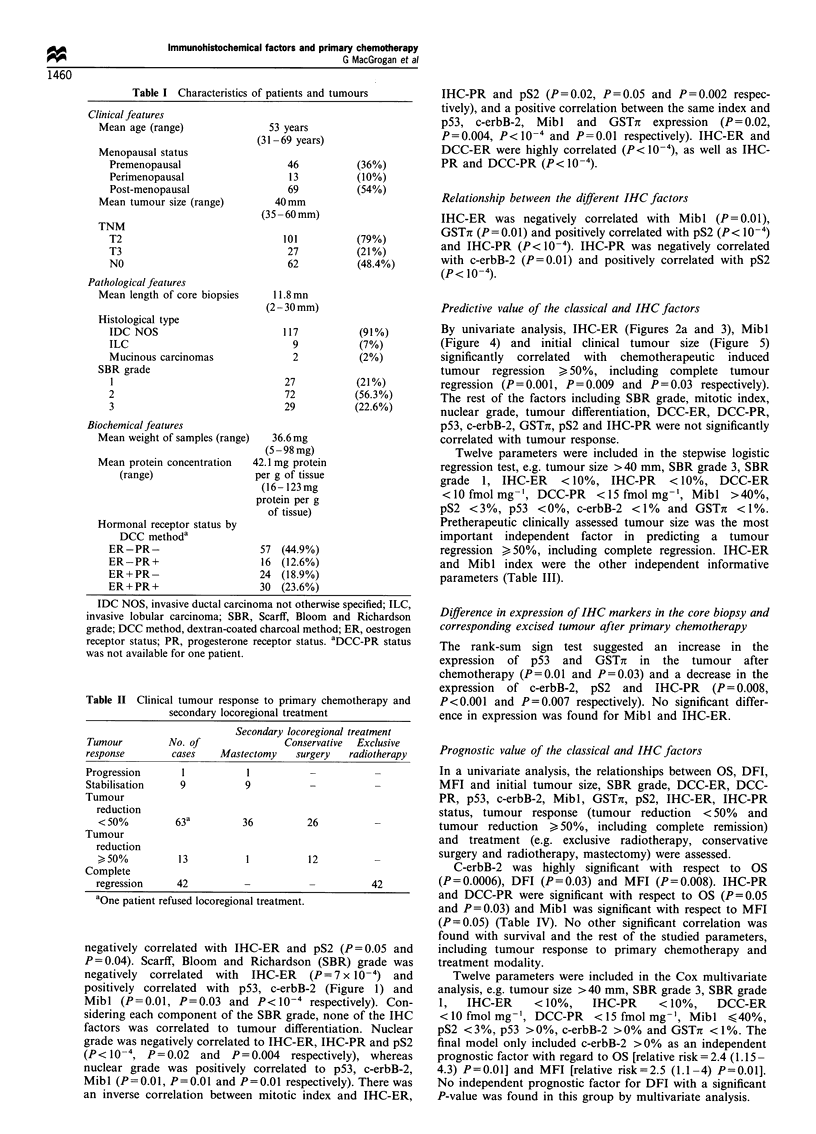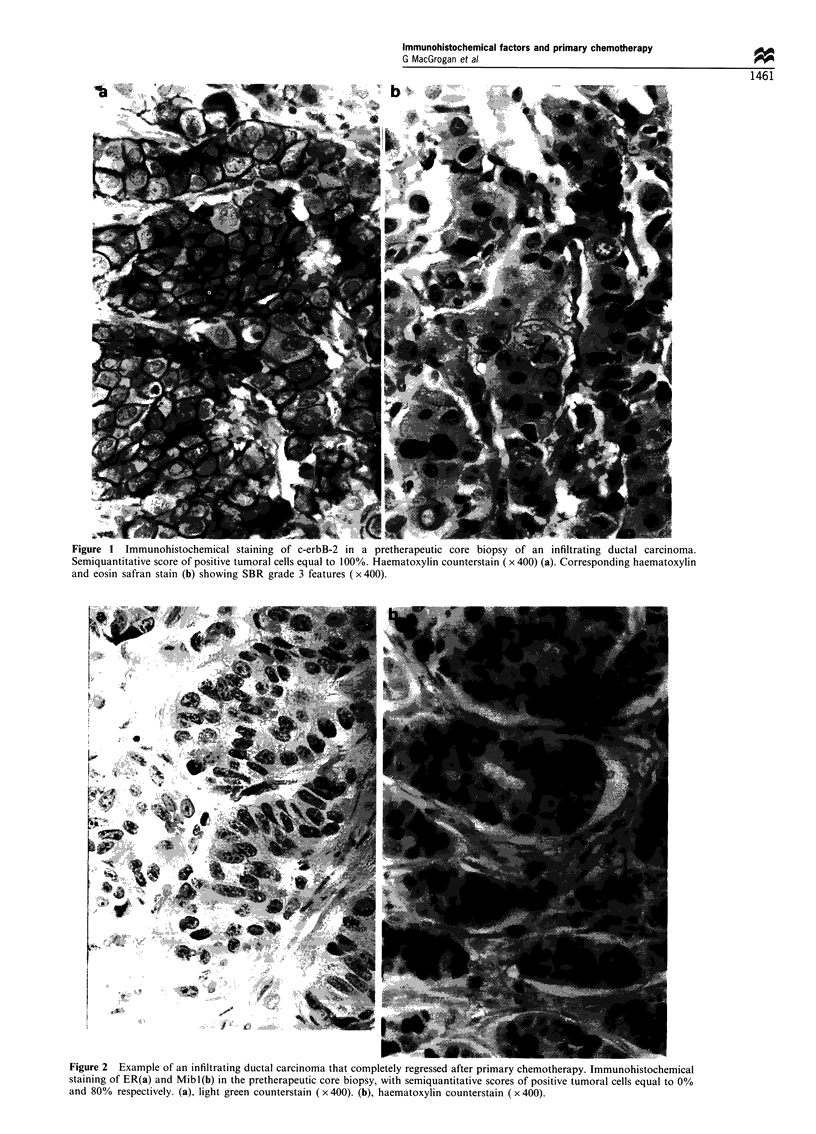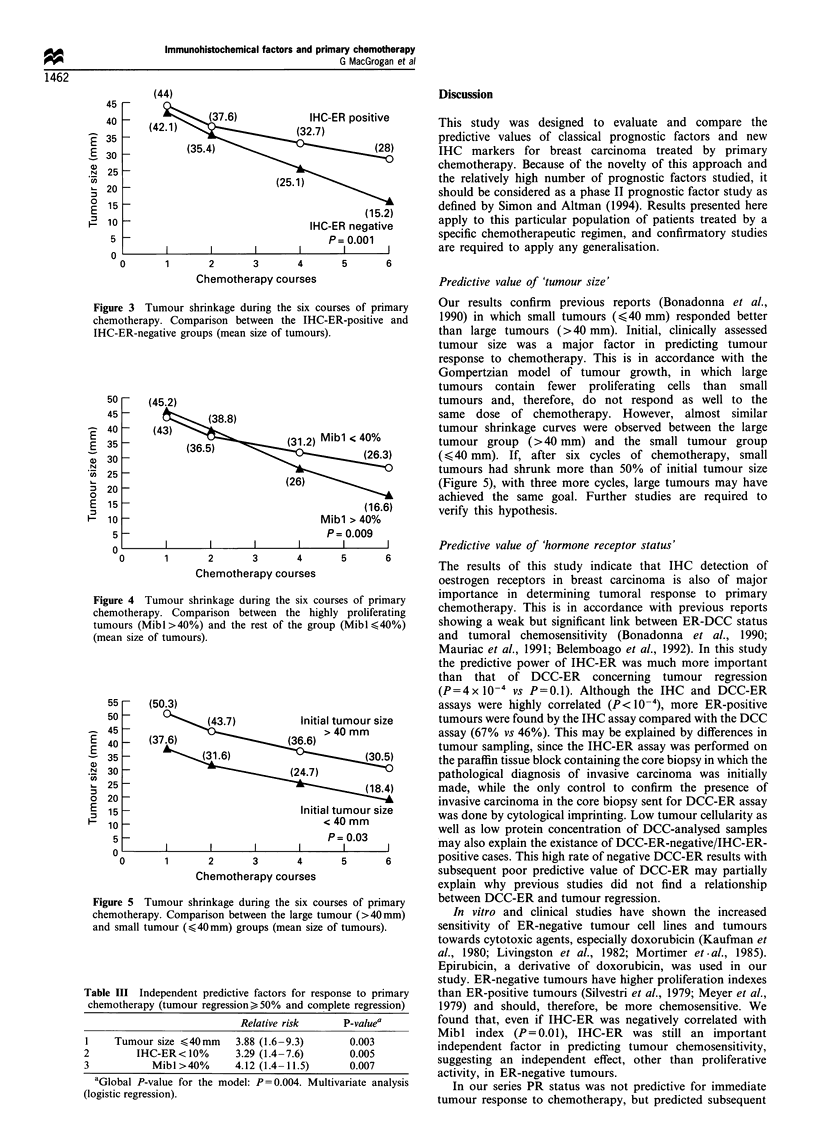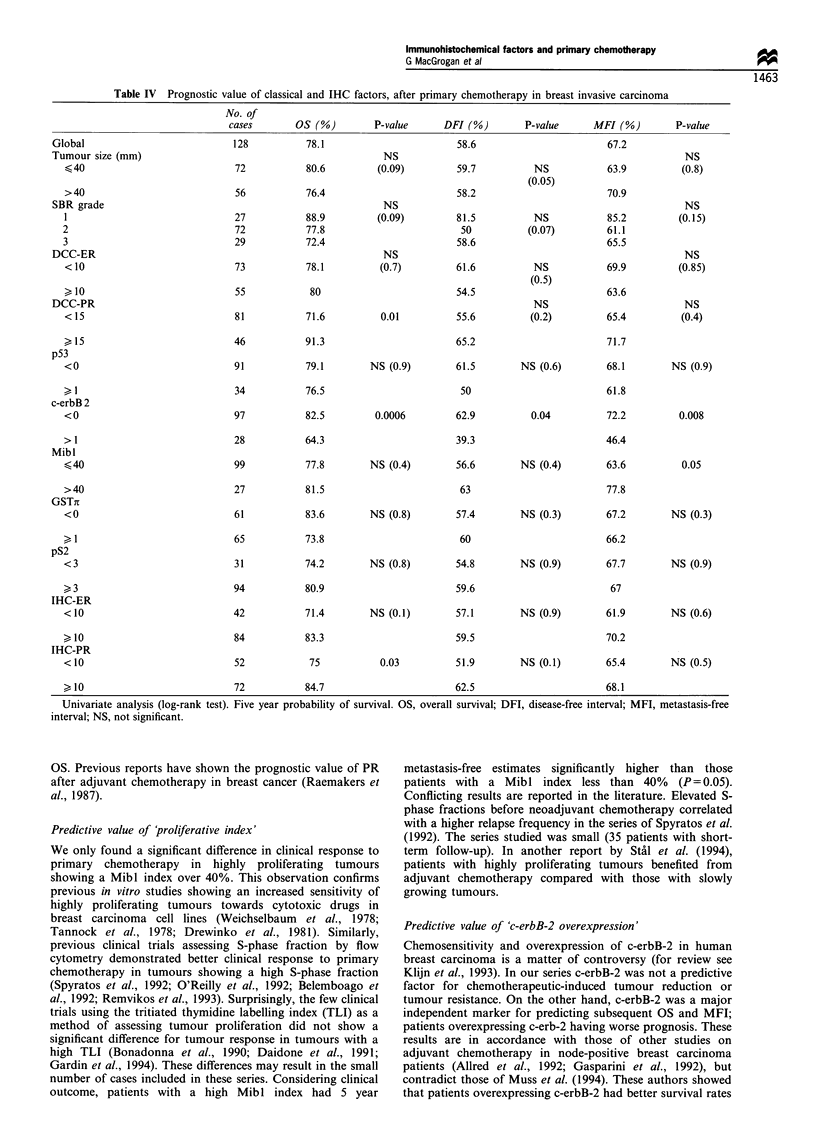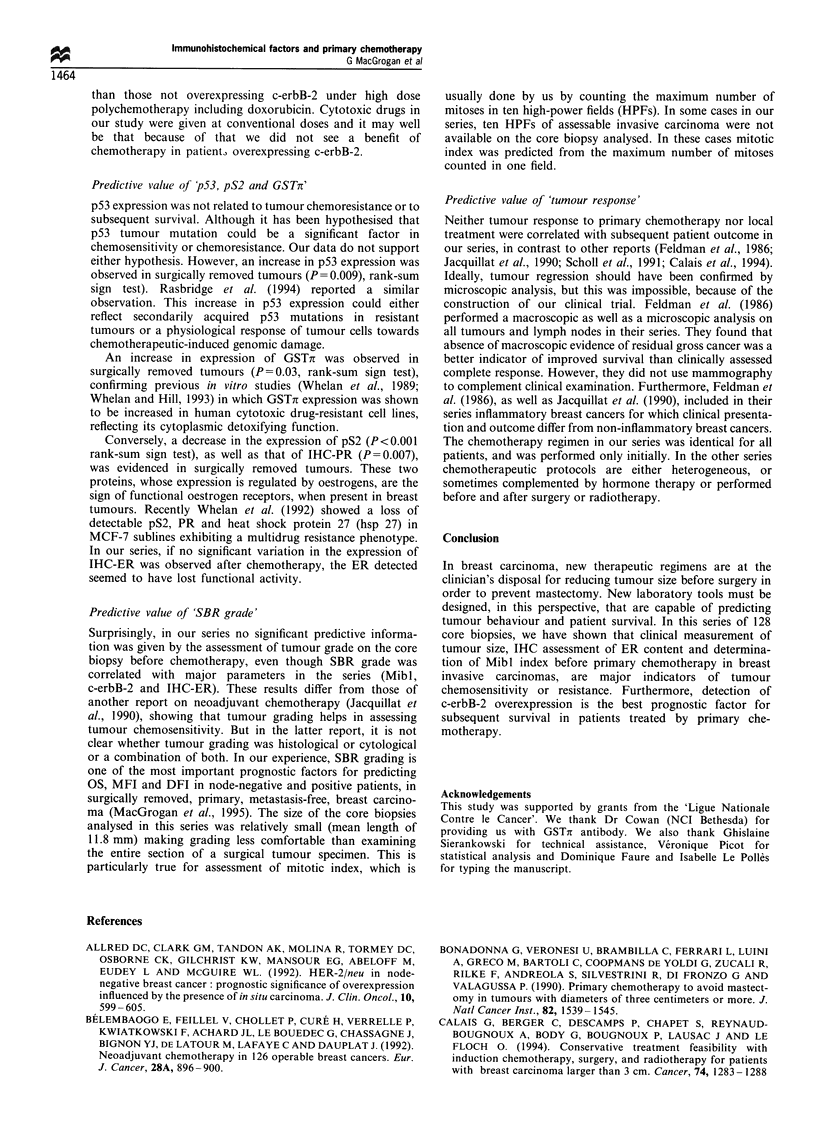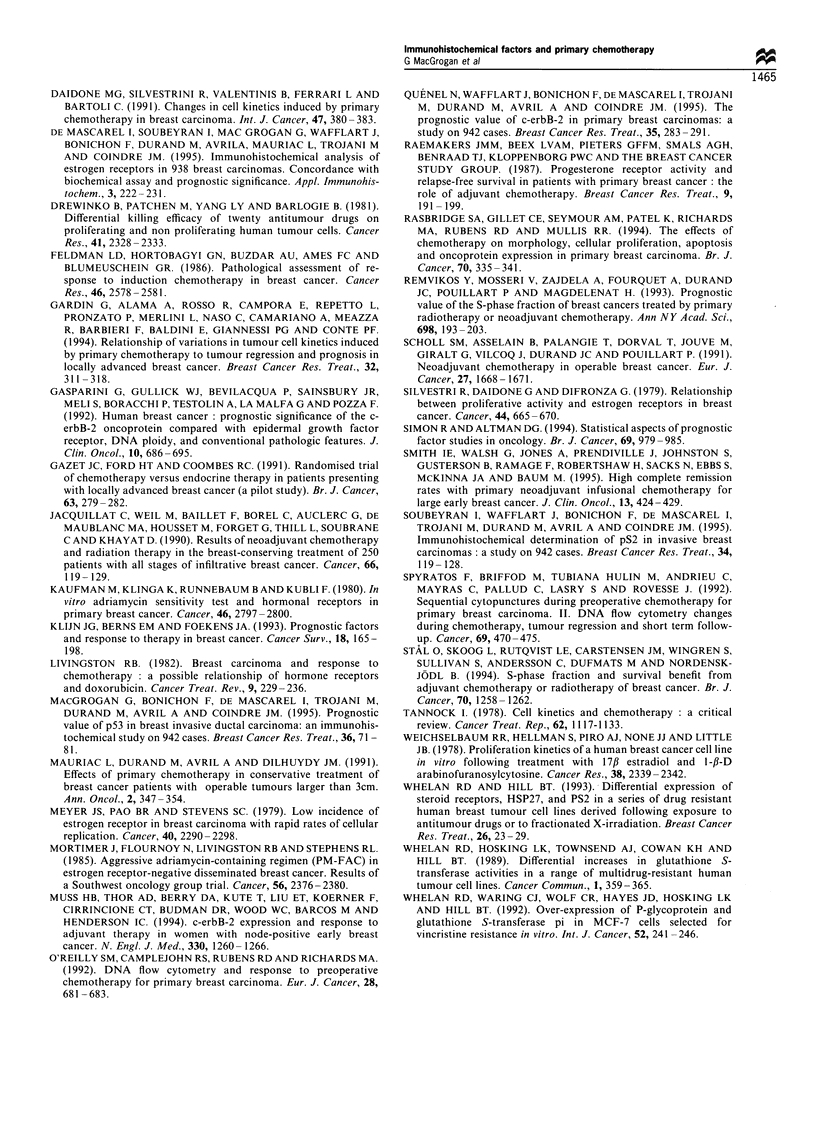# Primary chemotherapy in breast invasive carcinoma: predictive value of the immunohistochemical detection of hormonal receptors, p53, c-erbB-2, MiB1, pS2 and GST pi.

**DOI:** 10.1038/bjc.1996.565

**Published:** 1996-11

**Authors:** G. MacGrogan, L. Mauriac, M. Durand, F. Bonichon, M. Trojani, I. de Mascarel, J. M. Coindre

**Affiliations:** Institut Bergonié, Comprehensive Cancer Center, Bordeaux, France.

## Abstract

**Images:**


British Journal of Cancer (1996) 74, 1458-1465
© 1996 Stockton Press All rights reserved 0007-0920/96 $12.00

### Primary chemotherapy in breast invasive carcinoma: predictive value of the immunohistochemical detection of hormonal receptors, p53, c-erbB-2, MiB1, pS2 and GSTπ

G MacGrogan[1], L Mauriac[1], M Durand[1], F Bonichon[1], M Trojani[1], I de Mascarel[1] and JM Coindre[1,2]

[1]*Institut Bergonié, Comprehensive Cancer Center, 180 rue de Saint-Genès, 33076 Bordeaux, France;* [2]*Université de Bordeaux II, 140 rue Léo Saignat 33076, Bordeaux, France.*

**Summary** Primary chemotherapy in operable breast invasive carcinoma enables tumour reduction and conservative surgery. In order to search for one or more biological factors capable of predicting tumour behaviour under primary chemotherapy, and subsequent patient survival, an immunohistochemical study was performed with specific antibodies to p53, c-erbB-2 (Her-2/neu), Mibl (antiKi-67), pS2, GSTπ, oestrogen receptors (ERs) and progesterone receptors (PRs). Core biopsies, obtained before primary chemotherapy, were available from a series of 128 breast invasive carcinomas treated between January 1985 and April 1989, with a median follow-up of 93.3 months. Univariate statistical analysis showed that negative ER detection by immunohistochemistry (IHC) was highly correlated with chemosensitivity ($P=0.001$). A high percentage of Mibl-positive tumour cells (>40%), as well as initial tumour size less than 4 cm, were also correlated with tumour responsiveness to chemotherapy ($P=0.009$ and $P=0.03$). By multivariate analysis IHC-ER, Mibl and initial tumour size were independent predictors, the last parameter being the most important. Concerning subsequent patient survival, c-erbB-2 overexpression, as detected by IHC, was significant with respect to overall survival (OS) ($P=0.0006$), disease-free interval (DFI) ($P=0.03$) and metastasis-free interval (MFI) ($P=0.008$) by univariate analysis. Furthermore, c-erbB-2 was the major independent prognostic factor for OS and MFI by multivariate analysis.

**Keywords:** primary chemotherapy; breast neoplasm; oestrogen receptor; progesterone receptor; Mibl; c-erbB-2

Since 1990 several reports have shown the effectiveness of primary chemotherapy in breast conservative surgery of resectable tumours larger than 3 cm (Bonnadonna *et al.*, 1990; Gazet *et al.*, 1991; Mauriac *et al.*, 1991; Belembaogo *et al.*, 1992; Calais *et al.*, 1994; Smith *et al.*, 1995). This new therapeutic strategy, as well as those used in locally advanced breast disease, requires the validation of clinical and biological factors capable of predicting the tumoral response to cytotoxic therapy, and the subsequent clinical outcome. Until now most prognostic markers were designed and validated on primary surgery series of breast invasive carcinoma, complemented or not by adjuvant hormono- or chemotherapy. In these series it is sometimes difficult to grasp the meaning of the information provided by a prognostic factor in relation to the response to adjuvant therapy once the primary tumour has been removed. The primary chemotherapy regimens are elegant *in vivo* models where the informative value of a given tumour factor in relation to a certain cytotoxic drug can be assessed by simply analysing tumour shrinkage. Furthermore, the different criteria helping the clinician to predict patient outcome are somewhat different in the two therapeutic approaches, where one major information source, e.g. histological assessment of axillary lymph node involvement, is missing in the primary chemotherapy group. Pretherapeutic tumour core biopsies provide sufficient material to confirm the malignant and infiltrative nature of a breast tumour as well as to study and compare the predictive and prognostic values of different immunohistochemical factors.

At Bergonie Institute we have conducted a clinical trial on the effects of primary chemotherapy in conservative treatment of breast invasive carcinoma (Mauriac *et al.*, 1991). A set of 134 core biopsies was at our disposal to analyse different immunohistochemical factors including : the products of a tumour-suppressor gene, p53; an oncogene; c-*erb*B-2 (or Her-2/*neu*); a detoxifying agent, glutathione- *S*-transferase pi (GSTπ); a cell cycle nuclear protein, Ki67; an oestrogen-regulated protein, pS2 and finally, oestrogen and progesterone receptors. The aim of this study was to assess their predictive value for tumour response to primary chemotherapy and prognostic value for patient outcome and compare them with classical clinical and biological factors.

### Materials and methods

### *Patient selection*

Breast tumour core biopsies of 134 patients were retrieved from the files of the pathology department of Institute Bergonie and included in this immunohistochemical study. These patients belonged to the chemotherapy arm of a clinical trial on the effects of primary chemotherapy in conservative treatment of breast invasive carcinoma. The clinical trial was conducted at Bergonie Institute from January 1985 to April 1989 and included a total of 272 women (for details see Mauriac *et al.*, 1991). Briefly, the aim of this trial was to compare initial surgery and primary chemotherapy on primary metastasis-free operable breast tumours larger than 3 cm.

Before randomisation, two samples were obtained from each breast tumour. One sample was fixed in Bouin Hollande and embedded in paraffin before histological analysis. For the present study, six core biopsies of the initial 134 were excluded because of insufficient residual material. In the other sample oestrogen and progesterone receptor contents were determined by the dextran-coated charcoal method (DCC), with cut-off levels of 10 and 15 fmol $mg^{-1}$ of protein respectively.

Primary chemotherapy consisted of six courses, three with epirubicin, vincristine, methotrexate (EVM) followed by three with mitomycin C, thiotepa and vindesine (MTV). After

Correspondence: G MacGrogan
Received 16 October 1995; revised 23 April 1996; accepted 15 May 1996

completion of the sixth course, clinical examination and a radiographic mammogram were used to assess tumour regression. Locoregional treatment depended on this parameter.

Exclusive radiotherapy was performed in the case of complete regression. Conservative breast surgery with axillary lymph node dissection followed by radiotherapy were done in the cases of incomplete tumour regression with residual tumour measuring less than 2 cm in diameter. Mastectomy was performed in the remaining cases.

Histological examination was done on all excised tumours. Thus, 128 core biopsies and 86 excised tumours are analysed in this study.

*Immunohistochemical assay*

The IHC assays used in this study have been fully described elsewhere (Soubeyran *et al.*, 1995; de Mascarel *et al.*, 1995). Briefly, an antigen retrieval step was performed by heating tissue sections in a citrate buffer. Two staining methods were applied : a labelled streptavidin–biotin–peroxidase method (LSAB Kit, Dako, France) (p53, c-erbB-2, GSTπ, pS2) or an avidin–streptavidin–biotin peroxydase method (Strept ABC complex/HRP Duet Kit, Dako, France) (ER, PR, Mib1). The following primary antibodies were applied: p53 [mouse monoclonal DO7 (Dako, Trappes, France), dilution 1:100 in phosphate-buffered saline (PBS), 30 min at room temperature]; c-erbB-2 [rabbit polyclonal (Dako), dilution 1:600 in PBS, 10 min incubation at room temperature]; GSTπ [rabbit polyclonal (a kind gift from Dr K Cowan, NCI, Bethesda, MD, USA), dilution 1:3000 in PBS, 2 h incubation at room temperature]; pS2 [mouse monoclonal (CIS Bioindustries, France), dilution 1:10 in PBS, overnight incubation at room temperature]; Mib1 [mouse monoclonal antiKi67 (Immunotech, France), dilution 1:100 in PBS, 1 h incubation at room temperature]; ER [mouse monoclonal clone 1D5 (Dako), dilution 1:25 in PBS, 45 min incubation at room temperature]; PR [rat monoclonal clone PgR-ICA (Abbott Inc), dilution 1:10 in PBS, overnight incubation at room temperature].

Diaminobenzidine was used as chromogen. Haematoxylin was used as counterstain for the c-erbB-2, GSTπ, pS2 and the Mib1 assays, and light green was used as counterstain for the p53, ER and PR assays. Concerning the Mib1 assay, sections were predigested in 0.1% trypsin, 0.4% calcium chloride in PBS for 10 min before microwaving.

*Scoring system*

All the slides were scored by one of the authors (GMG). For p53, Mib1, ER and PR, nuclear staining of invasive tumour cells was scored as positive. For c-erbB-2, membranous staining of invasive tumour cells was scored as positive. For GSTπ and pS2, cytoplasmic staining of invasive tumour cells was scored as positive. The number of positive cells per tissue section was determined semiquantitively from 0% to 100%. The threshold for p53, c-erbB-2 and GSTπ positivity, was 1%; for pS2 positivity, 3%; and for IHC-ER and IHC-PR positivity, 10%. These optimal thresholds have already been determined in previous studies (MacGrogan *et al.*, 1995; Quénel *et al.*, 1995; Soubeyran *et al.*, 1995; de Mascarel *et al.*, 1995) to be the most informative for clinical outcome.

*Statistical analysis*

The chi-square test was used to investigate the significance of the relationship between the different IHC markers, expressed as dichotomised factors, and classical prognostic parameters, e.g. histological grade, hormonal receptor status, as well as the different IHC markers between themselves. The relationship between the IHC factors and patients' age as well as clinical tumour size was analysed by Student's *t*-test.

Differences in expression of IHC factors between the core biopsy and corresponding excised tumour after primary chemotherapy were studied using a non-parametric rank-sum sign test.

Relationship between the different factors and tumour regression was determined in a univariate analysis by the log-rank test using the Kaplan–Meier method. Interrelationship between the different predictive factors was determined by multivariate analysis using a logistic regression test. The variable to predict was tumour reduction $\geqslant 50\%$, including complete tumour remission. All factors were entered in the logistic regression analysis whatever their *P*-value by univariate analysis; but only those with a *P*-value $\leqslant 1\%$ were kept in the final model.

The log-rank test using the Kaplan–Meier method was again used to study the relationship between the different factors and prognosis expressed as 5 year probability of survival. A multivariate analysis using the Cox proportional hazard model permitted statistical evaluation of the different prognostic factors. All factors were entered in the Cox regression analysis whatever their *P*-value by univariate analysis; but only those with a *P*-value $\leqslant 1\%$ were kept in the final model.

Clinical size of the tumours was assessed before treatment, before the second and fourth courses of chemotherapy and at the sixth. Patient follow-up was done quarterly for 2 years, twice a year and finally yearly. For overall survival (OS), survival duration was calculated from the randomisation date to death, or the date they were last known alive. All causes of death were considered as events. For metastasis-free interval (MFI) and for disease-free interval (DFI), time to failure was computed from the randomisation date until metastasis or relapse, or the date they were last known to be disease-free respectively. For DFI, local failure and/or metastasis were considered as events. The cut-off date for the current analysis was 1 May 1995 with a median follow-up of 93.3 months. Univariate analyses for survival were performed using the log-rank tests and BMDP software, program 1L. Multivariate analyses were performed stepwise with the logistic regression or the Cox regression models using BMDP 2L.

### Results

Clinical, pathological and biological characteristics of this series are listed in Table I. Distribution of patients in treatment groups according to tumour response is shown in Table II.

*p53, c-erbB-2, Mib1, GSTπ, pS2, IHC-ER, IHC-PR expression in the series*

Eighty-four out of 126 analysed cases (67%) and 72 out of 124 analysed cases (58%) were respectively ER positive and PR positive by the IHC assay. Thirty-four (27%), 28 (22%) and 94 (75%) out of 125 analysed cases were respectively p53, c-erbB-2 and pS2 positive, and finally, 65 out of 126 analysed cases (52%) were GSTπ positive by the IHC assay.

One hundred and twenty-three out of 125 core biopsies contained Mib1-positive cells (97.7%). Mib1 positivity ranged from 3% to 90% of tumoral cells with a median value of 20%. A Mib1 index of 40%, corresponding to the 75th percentile in the group, was arbitrarily chosen as threshold at the beginning of the study to differentiate highly proliferating tumours from the rest of the group. Twenty-seven cases (21.4%) had more than 40% Mib1-positive tumour cells compared with 99 (78.6%) who had up to 40% Mib1 positivity.

*Relationship between the immunohistochemical factors and the classical prognostic parameters*

Age was respectively positively and negatively correlated with IHC-ER and GSTπ ($P<10^{-3}$ and $P=0.05$). Initial clinically assessed tumour size was respectively positively and

Table I Characteristics of patients and tumours

| | | |
|---|---|---|
| *Clinical features* | | |
| Mean age (range) | 53 years (31–69 years) | |
| Menopausal status | | |
| Premenopausal | 46 | (36%) |
| Perimenopausal | 13 | (10%) |
| Post-menopausal | 69 | (54%) |
| Mean tumour size (range) | 40 mm (35–60 mm) | |
| TNM | | |
| T2 | 101 | (79%) |
| T3 | 27 | (21%) |
| N0 | 62 | (48.4%) |
| *Pathological features* | | |
| Mean length of core biopsies | 11.8 mn (2–30 mm) | |
| Histological type | | |
| IDC NOS | 117 | (91%) |
| ILC | 9 | (7%) |
| Mucinous carcinomas | 2 | (2%) |
| SBR grade | | |
| 1 | 27 | (21%) |
| 2 | 72 | (56.3%) |
| 3 | 29 | (22.6%) |
| *Biochemical features* | | |
| Mean weight of samples (range) | 36.6 mg (5–98 mg) | |
| Mean protein concentration (range) | 42.1 mg protein per g of tissue (16–123 mg protein per g of tissue) | |
| Hormonal receptor status by DCC method[a] | | |
| ER–PR– | 57 (44.9%) | |
| ER–PR+ | 16 (12.6%) | |
| ER+PR– | 24 (18.9%) | |
| ER+PR+ | 30 (23.6%) | |

IDC NOS, invasive ductal carcinoma not otherwise specified; ILC, invasive lobular carcinoma; SBR, Scarff, Bloom and Richardson grade; DCC method, dextran-coated charcoal method; ER, oestrogen receptor status; PR, progesterone receptor status. [a]DCC-PR status was not available for one patient.

Table II Clinical tumour response to primary chemotherapy and secondary locoregional treatment

| *Tumour response* | *No. of cases* | *Secondary locoregional treatment* *Mastectomy* | *Conservative surgery* | *Exclusive radiotherapy* |
|---|---|---|---|---|
| Progression | 1 | 1 | – | – |
| Stabilisation | 9 | 9 | – | – |
| Tumour reduction <50% | 63[a] | 36 | 26 | – |
| Tumour reduction ⩾50% | 13 | 1 | 12 | – |
| Complete regression | 42 | – | – | 42 |

[a]One patient refused locoregional treatment.

negatively correlated with IHC-ER and pS2 ($P=0.05$ and $P=0.04$). Scarff, Bloom and Richardson (SBR) grade was negatively correlated with IHC-ER ($P=7\times10^{-4}$) and positively correlated with p53, c-erbB-2 (Figure 1) and Mib1 ($P=0.01$, $P=0.03$ and $P<10^{-4}$ respectively). Considering each component of the SBR grade, none of the IHC factors was correlated to tumour differentiation. Nuclear grade was negatively correlated to IHC-ER, IHC-PR and pS2 ($P<10^{-4}$, $P=0.02$ and $P=0.004$ respectively), whereas nuclear grade was positively correlated to p53, c-erbB-2, Mib1 ($P=0.01$, $P=0.01$ and $P=0.01$ respectively). There was an inverse correlation between mitotic index and IHC-ER, IHC-PR and pS2 ($P=0.02$, $P=0.05$ and $P=0.002$ respectively), and a positive correlation between the same index and p53, c-erbB-2, Mib1 and GST$\pi$ expression ($P=0.02$, $P=0.004$, $P<10^{-4}$ and $P=0.01$ respectively). IHC-ER and DCC-ER were highly correlated ($P<10^{-4}$), as well as IHC-PR and DCC-PR ($P<10^{-4}$).

### *Relationship between the different IHC factors*

IHC-ER was negatively correlated with Mib1 ($P=0.01$), GST$\pi$ ($P=0.01$) and positively correlated with pS2 ($P<10^{-4}$) and IHC-PR ($P<10^{-4}$). IHC-PR was negatively correlated with c-erbB-2 ($P=0.01$) and positively correlated with pS2 ($P<10^{-4}$).

### *Predictive value of the classical and IHC factors*

By univariate analysis, IHC-ER (Figures 2a and 3), Mib1 (Figure 4) and initial clinical tumour size (Figure 5) significantly correlated with chemotherapeutic induced tumour regression ⩾50%, including complete tumour regression ($P=0.001$, $P=0.009$ and $P=0.03$ respectively). The rest of the factors including SBR grade, mitotic index, nuclear grade, tumour differentiation, DCC-ER, DCC-PR, p53, c-erbB-2, GST$\pi$, pS2 and IHC-PR were not significantly correlated with tumour response.

Twelve parameters were included in the stepwise logistic regression test, e.g. tumour size >40 mm, SBR grade 3, SBR grade 1, IHC-ER <10%, IHC-PR <10%, DCC-ER <10 fmol mg$^{-1}$, DCC-PR <15 fmol mg$^{-1}$, Mib1 >40%, pS2 <3%, p53 <0%, c-erbB-2 <1% and GST$\pi$ <1%. Pretherapeutic clinically assessed tumour size was the most important independent factor in predicting a tumour regression ⩾50%, including complete regression. IHC-ER and Mib1 index were the other independent informative parameters (Table III).

### *Difference in expression of IHC markers in the core biopsy and corresponding excised tumour after primary chemotherapy*

The rank-sum sign test suggested an increase in the expression of p53 and GST$\pi$ in the tumour after chemotherapy ($P=0.01$ and $P=0.03$) and a decrease in the expression of c-erbB-2, pS2 and IHC-PR ($P=0.008$, $P<0.001$ and $P=0.007$ respectively). No significant difference in expression was found for Mib1 and IHC-ER.

### *Prognostic value of the classical and IHC factors*

In a univariate analysis, the relationships between OS, DFI, MFI and initial tumour size, SBR grade, DCC-ER, DCC-PR, p53, c-erbB-2, Mib1, GST$\pi$, pS2, IHC-ER, IHC-PR status, tumour response (tumour reduction <50% and tumour reduction ⩾50%, including complete remission) and treatment (e.g. exclusive radiotherapy, conservative surgery and radiotherapy, mastectomy) were assessed.

C-erbB-2 was highly significant with respect to OS ($P=0.0006$), DFI ($P=0.03$) and MFI ($P=0.008$). IHC-PR and DCC-PR were significant with respect to OS ($P=0.05$ and $P=0.03$) and Mib1 was significant with respect to MFI ($P=0.05$) (Table IV). No other significant correlation was found with survival and the rest of the studied parameters, including tumour response to primary chemotherapy and treatment modality.

Twelve parameters were included in the Cox multivariate analysis, e.g. tumour size >40 mm, SBR grade 3, SBR grade 1, IHC-ER <10%, IHC-PR <10%, DCC-ER <10 fmol mg$^{-1}$, DCC-PR <15 fmol mg$^{-1}$, Mib1 ⩽40%, pS2 <3%, p53 >0%, c-erbB-2 >0% and GST$\pi$ <1%. The final model only included c-erbB-2 >0% as an independent prognostic factor with regard to OS [relative risk = 2.4 (1.15–4.3) $P=0.01$] and MFI [relative risk = 2.5 (1.1–4) $P=0.01$]. No independent prognostic factor for DFI with a significant $P$-value was found in this group by multivariate analysis.

**Figure 1** Immunohistochemical staining of c-erbB-2 in a pretherapeutic core biopsy of an infiltrating ductal carcinoma. Semiquantitative score of positive tumoral cells equal to 100%. Haematoxylin counterstain (×400) (**a**). Corresponding haematoxylin and eosin safran stain (**b**) showing SBR grade 3 features (×400).

**Figure 2** Example of an infiltrating ductal carcinoma that completely regressed after primary chemotherapy. Immunohistochemical staining of ER(**a**) and Mib1(**b**) in the pretherapeutic core biopsy, with semiquantitative scores of positive tumoral cells equal to 0% and 80% respectively. (**a**), light green counterstain (×400). (**b**), haematoxylin counterstain (×400).

**Figure 3** Tumour shrinkage during the six courses of primary chemotherapy. Comparison between the IHC-ER-positive and IHC-ER-negative groups (mean size of tumours).

**Figure 4** Tumour shrinkage during the six courses of primary chemotherapy. Comparison between the highly proliferating tumours (Mibl >40%) and the rest of the group (Mibl ⩽40%) (mean size of tumours).

**Figure 5** Tumour shrinkage during the six courses of primary chemotherapy. Comparison between the large tumour (>40 mm) and small tumour (⩽40 mm) groups (mean size of tumours).

**Table III** Independent predictive factors for response to primary chemotherapy (tumour regression ⩾50% and complete regression)

| | | *Relative risk* | P-*value*[a] |
|---|---|---|---|
| 1 | Tumour size ⩽40 mm | 3.88 (1.6–9.3) | 0.003 |
| 2 | IHC-ER < 10% | 3.29 (1.4–7.6) | 0.005 |
| 3 | Mibl >40% | 4.12 (1.4–11.5) | 0.007 |

[a]Global $P$-value for the model: $P = 0.004$. Multivariate analysis (logistic regression).

### Discussion

This study was designed to evaluate and compare the predictive values of classical prognostic factors and new IHC markers for breast carcinoma treated by primary chemotherapy. Because of the novelty of this approach and the relatively high number of prognostic factors studied, it should be considered as a phase II prognostic factor study as defined by Simon and Altman (1994). Results presented here apply to this particular population of patients treated by a specific chemotherapeutic regimen, and confirmatory studies are required to apply any generalisation.

### *Predictive value of 'tumour size'*

Our results confirm previous reports (Bonadonna *et al.*, 1990) in which small tumours (⩽40 mm) responded better than large tumours (>40 mm). Initial, clinically assessed tumour size was a major factor in predicting tumour response to chemotherapy. This is in accordance with the Gompertzian model of tumour growth, in which large tumours contain fewer proliferating cells than small tumours and, therefore, do not respond as well to the same dose of chemotherapy. However, almost similar tumour shrinkage curves were observed between the large tumour group (>40 mm) and the small tumour group (⩽40 mm). If, after six cycles of chemotherapy, small tumours had shrunk more than 50% of initial tumour size (Figure 5), with three more cycles, large tumours may have achieved the same goal. Further studies are required to verify this hypothesis.

### *Predictive value of 'hormone receptor status'*

The results of this study indicate that IHC detection of oestrogen receptors in breast carcinoma is also of major importance in determining tumoral response to primary chemotherapy. This is in accordance with previous reports showing a weak but significant link between ER-DCC status and tumoral chemosensitivity (Bonadonna *et al.*, 1990; Mauriac *et al.*, 1991; Belemboago *et al.*, 1992). In this study the predictive power of IHC-ER was much more important than that of DCC-ER concerning tumour regression ($P = 4 \times 10^{-4}$ *vs* $P = 0.1$). Although the IHC and DCC-ER assays were highly correlated ($P < 10^{-4}$), more ER-positive tumours were found by the IHC assay compared with the DCC assay (67% *vs* 46%). This may be explained by differences in tumour sampling, since the IHC-ER assay was performed on the paraffin tissue block containing the core biopsy in which the pathological diagnosis of invasive carcinoma was initially made, while the only control to confirm the presence of invasive carcinoma in the core biopsy sent for DCC-ER assay was done by cytological imprinting. Low tumour cellularity as well as low protein concentration of DCC-analysed samples may also explain the existance of DCC-ER-negative/IHC-ER-positive cases. This high rate of negative DCC-ER results with subsequent poor predictive value of DCC-ER may partially explain why previous studies did not find a relationship between DCC-ER and tumour regression.

*In vitro* and clinical studies have shown the increased sensitivity of ER-negative tumour cell lines and tumours towards cytotoxic agents, especially doxorubicin (Kaufman *et al.*, 1980; Livingston *et al.*, 1982; Mortimer *et al.*, 1985). Epirubicin, a derivative of doxorubicin, was used in our study. ER-negative tumours have higher proliferation indexes than ER-positive tumours (Silvestri *et al.*, 1979; Meyer *et al.*, 1979) and should, therefore, be more chemosensitive. We found that, even if IHC-ER was negatively correlated with Mibl index ($P = 0.01$), IHC-ER was still an important independent factor in predicting tumour chemosensitivity, suggesting an independent effect, other than proliferative activity, in ER-negative tumours.

In our series PR status was not predictive for immediate tumour response to chemotherapy, but predicted subsequent

**Table IV** Prognostic value of classical and IHC factors, after primary chemotherapy in breast invasive carcinoma

| | *No. of cases* | *OS (%)* | P-*value* | *DFI (%)* | P-*value* | *MFI (%)* | P-*value* |
|---|---|---|---|---|---|---|---|
| Global | 128 | 78.1 | | 58.6 | | 67.2 | |
| Tumour size (mm) | | | NS | | | | NS |
| ≤40 | 72 | 80.6 | (0.09) | 59.7 | NS (0.05) | 63.9 | (0.8) |
| >40 | 56 | 76.4 | | 58.2 | | 70.9 | |
| SBR grade | | | NS | | | | NS |
| 1 | 27 | 88.9 | (0.09) | 81.5 | NS | 85.2 | (0.15) |
| 2 | 72 | 77.8 | | 50 | (0.07) | 61.1 | |
| 3 | 29 | 72.4 | | 58.6 | | 65.5 | |
| DCC-ER | | | NS | | | | NS |
| <10 | 73 | 78.1 | (0.7) | 61.6 | NS (0.5) | 69.9 | (0.85) |
| ≥10 | 55 | 80 | | 54.5 | | 63.6 | |
| DCC-PR | | | | | NS | | NS |
| <15 | 81 | 71.6 | 0.01 | 55.6 | (0.2) | 65.4 | (0.4) |
| ≥15 | 46 | 91.3 | | 65.2 | | 71.7 | |
| p53 | | | | | | | |
| <0 | 91 | 79.1 | NS (0.9) | 61.5 | NS (0.6) | 68.1 | NS (0.9) |
| ≥1 | 34 | 76.5 | | 50 | | 61.8 | |
| c-erbB2 | | | | | | | |
| <0 | 97 | 82.5 | 0.0006 | 62.9 | 0.04 | 72.2 | 0.008 |
| >1 | 28 | 64.3 | | 39.3 | | 46.4 | |
| Mib1 | | | | | | | |
| ≤40 | 99 | 77.8 | NS (0.4) | 56.6 | NS (0.4) | 63.6 | 0.05 |
| >40 | 27 | 81.5 | | 63 | | 77.8 | |
| GSTπ | | | | | | | |
| <0 | 61 | 83.6 | NS (0.8) | 57.4 | NS (0.3) | 67.2 | NS (0.3) |
| ≥1 | 65 | 73.8 | | 60 | | 66.2 | |
| pS2 | | | | | | | |
| <3 | 31 | 74.2 | NS (0.8) | 54.8 | NS (0.9) | 67.7 | NS (0.9) |
| ≥3 | 94 | 80.9 | | 59.6 | | 67 | |
| IHC-ER | | | | | | | |
| <10 | 42 | 71.4 | NS (0.1) | 57.1 | NS (0.9) | 61.9 | NS (0.6) |
| ≥10 | 84 | 83.3 | | 59.5 | | 70.2 | |
| IHC-PR | | | | | | | |
| <10 | 52 | 75 | 0.03 | 51.9 | NS (0.1) | 65.4 | NS (0.5) |
| ≥10 | 72 | 84.7 | | 62.5 | | 68.1 | |

Univariate analysis (log-rank test). Five year probability of survival. OS, overall survival; DFI, disease-free interval; MFI, metastasis-free interval; NS, not significant.

OS. Previous reports have shown the prognostic value of PR after adjuvant chemotherapy in breast cancer (Raemakers *et al.*, 1987).

*Predictive value of 'proliferative index'*

We only found a significant difference in clinical response to primary chemotherapy in highly proliferating tumours showing a Mib1 index over 40%. This observation confirms previous *in vitro* studies showing an increased sensitivity of highly proliferating tumours towards cytotoxic drugs in breast carcinoma cell lines (Weichselbaum *et al.*, 1978; Tannock *et al.*, 1978; Drewinko *et al.*, 1981). Similarly, previous clinical trials assessing S-phase fraction by flow cytometry demonstrated better clinical response to primary chemotherapy in tumours showing a high S-phase fraction (Spyratos *et al.*, 1992; O'Reilly *et al.*, 1992; Belemboago *et al.*, 1992; Remvikos *et al.*, 1993). Surprisingly, the few clinical trials using the tritiated thymidine labelling index (TLI) as a method of assessing tumour proliferation did not show a significant difference for tumour response in tumours with a high TLI (Bonadonna *et al.*, 1990; Daidone *et al.*, 1991; Gardin *et al.*, 1994). These differences may result in the small number of cases included in these series. Considering clinical outcome, patients with a high Mib1 index had 5 year metastasis-free estimates significantly higher than those patients with a Mib1 index less than 40% ($P=0.05$). Conflicting results are reported in the literature. Elevated S-phase fractions before neoadjuvant chemotherapy correlated with a higher relapse frequency in the series of Spyratos *et al.* (1992). The series studied was small (35 patients with short-term follow-up). In another report by Stål *et al.* (1994), patients with highly proliferating tumours benefited from adjuvant chemotherapy compared with those with slowly growing tumours.

*Predictive value of 'c-erbB-2 overexpression'*

Chemosensitivity and overexpression of c-erbB-2 in human breast carcinoma is a matter of controversy (for review see Klijn *et al.*, 1993). In our series c-erbB-2 was not a predictive factor for chemotherapeutic-induced tumour reduction or tumour resistance. On the other hand, c-erbB-2 was a major independent marker for predicting subsequent OS and MFI; patients overexpressing c-erb-2 having worse prognosis. These results are in accordance with those of other studies on adjuvant chemotherapy in node-positive breast carcinoma patients (Allred *et al.*, 1992; Gasparini *et al.*, 1992), but contradict those of Muss *et al.* (1994). These authors showed that patients overexpressing c-erbB-2 had better survival rates than those not overexpressing c-erbB-2 under high dose polychemotherapy including doxorubicin. Cytotoxic drugs in our study were given at conventional doses and it may well be that because of that we did not see a benefit of chemotherapy in patients overexpressing c-erbB-2.

*Predictive value of 'p53, pS2 and GSTπ'*

p53 expression was not related to tumour chemoresistance or to subsequent survival. Although it has been hypothesised that p53 tumour mutation could be a significant factor in chemosensitivity or chemoresistance. Our data do not support either hypothesis. However, an increase in p53 expression was observed in surgically removed tumours ($P = 0.009$), rank-sum sign test). Rasbridge *et al.* (1994) reported a similar observation. This increase in p53 expression could either reflect secondarily acquired p53 mutations in resistant tumours or a physiological response of tumour cells towards chemotherapeutic-induced genomic damage.

An increase in expression of GSTπ was observed in surgically removed tumours ($P = 0.03$, rank-sum sign test), confirming previous *in vitro* studies (Whelan *et al.*, 1989; Whelan and Hill, 1993) in which GSTπ expression was shown to be increased in human cytotoxic drug-resistant cell lines, reflecting its cytoplasmic detoxifying function.

Conversely, a decrease in the expression of pS2 ($P < 0.001$ rank-sum sign test), as well as that of IHC-PR ($P = 0.007$), was evidenced in surgically removed tumours. These two proteins, whose expression is regulated by oestrogens, are the sign of functional oestrogen receptors, when present in breast tumours. Recently Whelan *et al.* (1992) showed a loss of detectable pS2, PR and heat shock protein 27 (hsp 27) in MCF-7 sublines exhibiting a multidrug resistance phenotype. In our series, if no significant variation in the expression of IHC-ER was observed after chemotherapy, the ER detected seemed to have lost functional activity.

*Predictive value of 'SBR grade'*

Surprisingly, in our series no significant predictive information was given by the assessment of tumour grade on the core biopsy before chemotherapy, even though SBR grade was correlated with major parameters in the series (Mibl, c-erbB-2 and IHC-ER). These results differ from those of another report on neoadjuvant chemotherapy (Jacquillat *et al.*, 1990), showing that tumour grading helps in assessing tumour chemosensitivity. But in the latter report, it is not clear whether tumour grading was histological or cytological or a combination of both. In our experience, SBR grading is one of the most important prognostic factors for predicting OS, MFI and DFI in node-negative and positive patients, in surgically removed, primary, metastasis-free, breast carcinoma (MacGrogan *et al.*, 1995). The size of the core biopsies analysed in this series was relatively small (mean length of 11.8 mm) making grading less comfortable than examining the entire section of a surgical tumour specimen. This is particularly true for assessment of mitotic index, which is usually done by us by counting the maximum number of mitoses in ten high-power fields (HPFs). In some cases in our series, ten HPFs of assessable invasive carcinoma were not available on the core biopsy analysed. In these cases mitotic index was predicted from the maximum number of mitoses counted in one field.

*Predictive value of 'tumour response'*

Neither tumour response to primary chemotherapy nor local treatment were correlated with subsequent patient outcome in our series, in contrast to other reports (Feldman *et al.*, 1986; Jacquillat *et al.*, 1990; Scholl *et al.*, 1991; Calais *et al.*, 1994). Ideally, tumour regression should have been confirmed by microscopic analysis, but this was impossible, because of the construction of our clinical trial. Feldman *et al.* (1986) performed a macroscopic as well as a microscopic analysis on all tumours and lymph nodes in their series. They found that absence of macroscopic evidence of residual gross cancer was a better indicator of improved survival than clinically assessed complete response. However, they did not use mammography to complement clinical examination. Furthermore, Feldman *et al.* (1986), as well as Jacquillat *et al.* (1990), included in their series inflammatory breast cancers for which clinical presentation and outcome differ from non-inflammatory breast cancers. The chemotherapy regimen in our series was identical for all patients, and was performed only initially. In the other series chemotherapeutic protocols are either heterogeneous, or sometimes complemented by hormone therapy or performed before and after surgery or radiotherapy.

### Conclusion

In breast carcinoma, new therapeutic regimens are at the clinician's disposal for reducing tumour size before surgery in order to prevent mastectomy. New laboratory tools must be designed, in this perspective, that are capable of predicting tumour behaviour and patient survival. In this series of 128 core biopsies, we have shown that clinical measurement of tumour size, IHC assessment of ER content and determination of Mibl index before primary chemotherapy in breast invasive carcinomas, are major indicators of tumour chemosensitivity or resistance. Furthermore, detection of c-erbB-2 overexpression is the best prognostic factor for subsequent survival in patients treated by primary chemotherapy.

**Acknowledgements**
This study was supported by grants from the 'Ligue Nationale Contre le Cancer'. We thank Dr Cowan (NCI Bethesda) for providing us with GSTπ antibody. We also thank Ghislaine Sierankowski for technical assistance, Véronique Picot for statistical analysis and Dominique Faure and Isabelle Le Pollès for typing the manuscript.

### References

ALLRED DC, CLARK GM, TANDON AK, MOLINA R, TORMEY DC, OSBORNE CK, GILCHRIST KW, MANSOUR EG, ABELOFF M, EUDEY L AND MCGUIRE WL. (1992). HER-2/*neu* in node-negative breast cancer : prognostic significance of overexpression influenced by the presence of *in situ* carcinoma. *J. Clin. Oncol.*, **10**, 599–605.

BÉLEMBAOGO E, FEILLEL V, CHOLLET P, CURÉ H, VERRELLE P, KWIATKOWSKI F, ACHARD JL, LE BOUEDEC G, CHASSAGNE J, BIGNON YJ, DE LATOUR M, LAFAYE C AND DAUPLAT J. (1992). Neoadjuvant chemotherapy in 126 operable breast cancers. *Eur. J. Cancer*, **28A**, 896–900.

BONADONNA G, VERONESI U, BRAMBILLA C, FERRARI L, LUINI A, GRECO M, BARTOLI C, COOPMANS DE YOLDI G, ZUCALI R, RILKE F, ANDREOLA S, SILVESTRINI R, DI FRONZO G AND VALAGUSSA P. (1990). Primary chemotherapy to avoid mastectomy in tumours with diameters of three centimeters or more. *J. Natl Cancer Inst.*, **82**, 1539–1545.

CALAIS G, BERGER C, DESCAMPS P, CHAPET S, REYNAUD-BOUGNOUX A, BODY G, BOUGNOUX P, LAUSAC J AND LE FLOCH O. (1994). Conservative treatment feasibility with induction chemotherapy, surgery, and radiotherapy for patients with breast carcinoma larger than 3 cm. *Cancer*, **74**, 1283–1288

DAIDONE MG, SILVESTRINI R, VALENTINIS B, FERRARI L AND BARTOLI C. (1991). Changes in cell kinetics induced by primary chemotherapy in breast carcinoma. *Int. J. Cancer*, **47,** 380–383.

DE MASCAREL I, SOUBEYRAN I, MAC GROGAN G, WAFFLART J, BONICHON F, DURAND M, AVRILA, MAURIAC L, TROJANI M AND COINDRE JM. (1995). Immunohistochemical analysis of estrogen receptors in 938 breast carcinomas. Concordance with biochemical assay and prognostic significance. *Appl. Immunohistochem.*, **3,** 222–231.

DREWINKO B, PATCHEN M, YANG LY AND BARLOGIE B. (1981). Differential killing efficacy of twenty antitumour drugs on proliferating and non proliferating human tumour cells. *Cancer Res.*, **41,** 2328–2333.

FELDMAN LD, HORTOBAGYI GN, BUZDAR AU, AMES FC AND BLUMEUSCHEIN GR. (1986). Pathological assessment of response to induction chemotherapy in breast cancer. *Cancer Res.*, **46,** 2578–2581.

GARDIN G, ALAMA A, ROSSO R, CAMPORA E, REPETTO L, PRONZATO P, MERLINI L, NASO C, CAMARIANO A, MEAZZA R, BARBIERI F, BALDINI E, GIANNESSI PG AND CONTE PF. (1994). Relationship of variations in tumour cell kinetics induced by primary chemotherapy to tumour regression and prognosis in locally advanced breast cancer. *Breast Cancer Res. Treat.*, **32,** 311–318.

GASPARINI G, GULLICK WJ, BEVILACQUA P, SAINSBURY JR, MELI S, BORACCHI P, TESTOLIN A, LA MALFA G AND POZZA F. (1992). Human breast cancer : prognostic significance of the c-erbB-2 oncoprotein compared with epidermal growth factor receptor, DNA ploidy, and conventional pathologic features. *J. Clin. Oncol.*, **10,** 686–695.

GAZET JC, FORD HT AND COOMBES RC. (1991). Randomised trial of chemotherapy versus endocrine therapy in patients presenting with locally advanced breast cancer (a pilot study). *Br. J. Cancer*, **63,** 279–282.

JACQUILLAT C, WEIL M, BAILLET F, BOREL C, AUCLERC G, DE MAUBLANC MA, HOUSSET M, FORGET G, THILL L, SOUBRANE C AND KHAYAT D. (1990). Results of neoadjuvant chemotherapy and radiation therapy in the breast-conserving treatment of 250 patients with all stages of infiltrative breast cancer. *Cancer*, **66,** 119–129.

KAUFMAN M, KLINGA K, RUNNEBAUM B AND KUBLI F. (1980). *In vitro* adriamycin sensitivity test and hormonal receptors in primary breast cancer. *Cancer*, **46,** 2797–2800.

KLIJN JG, BERNS EM AND FOEKENS JA. (1993). Prognostic factors and response to therapy in breast cancer. *Cancer Surv.*, **18,** 165–198.

LIVINGSTON RB. (1982). Breast carcinoma and response to chemotherapy : a possible relationship of hormone receptors and doxorubicin. *Cancer Treat. Rev.*, **9,** 229–236.

MACGROGAN G, BONICHON F, DE MASCAREL I, TROJANI M, DURAND M, AVRIL A AND COINDRE JM. (1995). Prognostic value of p53 in breast invasive ductal carcinoma: an immunohistochemical study on 942 cases. *Breast Cancer Res. Treat.*, **36,** 71–81.

MAURIAC L, DURAND M, AVRIL A AND DILHUYDY JM. (1991). Effects of primary chemotherapy in conservative treatment of breast cancer patients with operable tumours larger than 3cm. *Ann. Oncol.*, **2,** 347–354.

MEYER JS, PAO BR AND STEVENS SC. (1979). Low incidence of estrogen receptor in breast carcinoma with rapid rates of cellular replication. *Cancer*, **40,** 2290–2298.

MORTIMER J, FLOURNOY N, LIVINGSTON RB AND STEPHENS RL. (1985). Aggressive adriamycin-containing regimen (PM-FAC) in estrogen receptor-negative disseminated breast cancer. Results of a Southwest oncology group trial. *Cancer*, **56,** 2376–2380.

MUSS HB, THOR AD, BERRY DA, KUTE T, LIU ET, KOERNER F, CIRRINCIONE CT, BUDMAN DR, WOOD WC, BARCOS M AND HENDERSON IC. (1994). c-erbB-2 expression and response to adjuvant therapy in women with node-positive early breast cancer. *N. Engl. J. Med.*, **330,** 1260–1266.

O'REILLY SM, CAMPLEJOHN RS, RUBENS RD AND RICHARDS MA. (1992). DNA flow cytometry and response to preoperative chemotherapy for primary breast carcinoma. *Eur. J. Cancer*, **28,** 681–683.

QUÉNEL N, WAFFLART J, BONICHON F, DE MASCAREL I, TROJANI M, DURAND M, AVRIL A AND COINDRE JM. (1995). The prognostic value of c-erbB-2 in primary breast carcinomas: a study on 942 cases. *Breast Cancer Res. Treat.*, **35,** 283–291.

RAEMAKERS JMM, BEEX LVAM, PIETERS GFFM, SMALS AGH, BENRAAD TJ, KLOPPENBORG PWC AND THE BREAST CANCER STUDY GROUP. (1987). Progesterone receptor activity and relapse-free survival in patients with primary breast cancer : the role of adjuvant chemotherapy. *Breast Cancer Res. Treat.*, **9,** 191–199.

RASBRIDGE SA, GILLET CE, SEYMOUR AM, PATEL K, RICHARDS MA, RUBENS RD AND MULLIS RR. (1994). The effects of chemotherapy on morphology, cellular proliferation, apoptosis and oncoprotein expression in primary breast carcinoma. *Br. J. Cancer*, **70,** 335–341.

REMVIKOS Y, MOSSERI V, ZAJDELA A, FOURQUET A, DURAND JC, POUILLART P AND MAGDELENAT H. (1993). Prognostic value of the S-phase fraction of breast cancers treated by primary radiotherapy or neoadjuvant chemotherapy. *Ann NY Acad. Sci.*, **698,** 193–203.

SCHOLL SM, ASSELAIN B, PALANGIE T, DORVAL T, JOUVE M, GIRALT G, VILCOQ J, DURAND JC AND POUILLART P. (1991). Neoadjuvant chemotherapy in operable breast cancer. *Eur. J. Cancer*, **27,** 1668–1671.

SILVESTRI R, DAIDONE G AND DIFRONZA G. (1979). Relationship between proliferative activity and estrogen receptors in breast cancer. *Cancer*, **44,** 665–670.

SIMON R AND ALTMAN DG. (1994). Statistical aspects of prognostic factor studies in oncology. *Br. J. Cancer*, **69,** 979–985.

SMITH IE, WALSH G, JONES A, PRENDIVILLE J, JOHNSTON S, GUSTERSON B, RAMAGE F, ROBERTSHAW H, SACKS N, EBBS S, MCKINNA JA AND BAUM M. (1995). High complete remission rates with primary neoadjuvant infusional chemotherapy for large early breast cancer. *J. Clin. Oncol.*, **13,** 424–429.

SOUBEYRAN I, WAFFLART J, BONICHON F, DE MASCAREL I, TROJANI M, DURAND M, AVRIL A AND COINDRE JM. (1995). Immunohistochemical determination of pS2 in invasive breast carcinomas : a study on 942 cases. *Breast Cancer Res. Treat.*, **34,** 119–128.

SPYRATOS F, BRIFFOD M, TUBIANA HULIN M, ANDRIEU C, MAYRAS C, PALLUD C, LASRY S AND ROVESSE J. (1992). Sequential cytopunctures during preoperative chemotherapy for primary breast carcinoma. II. DNA flow cytometry changes during chemotherapy, tumour regression and short term follow-up. *Cancer*, **69,** 470–475.

STÅL O, SKOOG L, RUTQVIST LE, CARSTENSEN JM, WINGREN S, SULLIVAN S, ANDERSSON C, DUFMATS M AND NORDENSKJÖDL B. (1994). S-phase fraction and survival benefit from adjuvant chemotherapy or radiotherapy of breast cancer. *Br. J. Cancer*, **70,** 1258–1262.

TANNOCK I. (1978). Cell kinetics and chemotherapy : a critical review. *Cancer Treat. Rep.*, **62,** 1117-1133.

WEICHSELBAUM RR, HELLMAN S, PIRO AJ, NONE JJ AND LITTLE JB. (1978). Proliferation kinetics of a human breast cancer cell line *in vitro* following treatment with 17$\beta$ estradiol and 1-$\beta$-D arabinofuranosylcytosine. *Cancer Res.*, **38,** 2339–2342.

WHELAN RD AND HILL BT. (1993). Differential expression of steroid receptors, HSP27, and PS2 in a series of drug resistant human breast tumour cell lines derived following exposure to antitumour drugs or to fractionated X-irradiation. *Breast Cancer Res. Treat.*, **26,** 23–29.

WHELAN RD, HOSKING LK, TOWNSEND AJ, COWAN KH AND HILL BT. (1989). Differential increases in glutathione *S*-transferase activities in a range of multidrug-resistant human tumour cell lines. *Cancer Commun.*, **1,** 359–365.

WHELAN RD, WARING CJ, WOLF CR, HAYES JD, HOSKING LK AND HILL BT. (1992). Over-expression of P-glycoprotein and glutathione *S*-transferase pi in MCF-7 cells selected for vincristine resistance *in vitro*. *Int. J. Cancer*, **52,** 241–246.